# *SIRT5* Regulates Lipid Deposition in Goat Preadipocytes via PI3K-Akt and MAPK Signaling Pathways

**DOI:** 10.3390/ani15071072

**Published:** 2025-04-07

**Authors:** Haiyang Li, Wenli Yao, Changheng Yang, Wenyang Zhang, Yong Wang, Yaqiu Lin, Zhanyu Du, Changhui Zhang, Lian Huang, Ming Zhang, Huaigong Fan, Jiangjiang Zhu, Hua Xiang

**Affiliations:** 1 Qinghai-Tibetan Plateau Animal Genetic Resource Reservation and Utilization Key Laboratory of Sichuan Province, Southwest Minzu University, Chengdu 610041, China; haiyang6715@163.com (H.L.); yaowenli1999@163.com (W.Y.); yangch10282021@163.com (C.Y.); zhangwenyang6588@163.com (W.Z.); wangyong010101@hotmail.com (Y.W.); linyq1999@163.com (Y.L.); yuzhan.du@outlook.com (Z.D.); zhangchanghui0074@163.com (C.Z.); kin8248806@163.com (L.H.); zhangmingcoming@163.com (M.Z.); zhujiang4656@hotmail.com (J.Z.); 2 Key Laboratory of Qinghai-Tibetan Plateau Animal Genetic Resource Reservation and Utilization, Ministry of Education, Southwest Minzu University, Chengdu 610041, China; 3 Sichuan Guonong Tianfu Agricultural Development Co., Ltd., Chengdu 611441, China; schg5512938@163.com; 4 Institute of Qinghai-Tibetan Plateau, Southwest Minzu University, Chengdu 610041, China

**Keywords:** IMF, intramuscular adipocyte, *SIRT5*, RNA-seq, MAPK

## Abstract

Silent Information Regulator 5 (*SIRT5*) has been established as a crucial regulator of cellular alanylation modification. Furthermore, accumulating evidence suggests that *SIRT5* plays a significant regulatory role in key metabolic pathways, including glycolysis, the tricarboxylic acid (TCA) cycle, and fatty acid oxidation, all of which are closely associated with cellular lipid metabolism. Despite these advancements, the specific role of *SIRT5* in regulating intramuscular fat (IMF) deposition in goats, as well as the underlying molecular mechanisms, remains largely unexplored. In this study, we cloned the complete coding sequence of the goat *SIRT5* gene and, through amino acid sequence alignment, demonstrated its closest phylogenetic relationship with sheep. Additionally, we characterized the higher expression of *SIRT5* during the differentiation of goat intramuscular precursor adipocytes. The silencing of *SIRT5* by siRNA-mediated knockdown significantly upregulated the expression of lipogenesis-related genes and enhanced lipid deposition in goat intramuscular preadipocytes. Concurrently, *SIRT5* deficiency led to the inhibition of cell proliferation and a marked reduction in apoptosis. Interestingly, although overexpression of *SIRT5* promoted cell proliferation, it did not significantly alter lipid deposition in goat intramuscular precursor adipocytes. RNA sequencing (RNA-seq) analysis identified a total of 106 differentially expressed genes (DEGs) following *SIRT5* silencing in goat preadipocytes, predominantly involved in the Focal adhesion, HIF-1, PI3K-Akt, and MAPK signaling pathways by KEGG pathway enrichment analysis. Notably, we successfully reversed the phenotypic effects observed in *SIRT5* knockdown goat precursor adipocytes by inhibiting the PI3K-Akt and MAPK signaling pathways using the AKT inhibitor LY294002 and the p38 MAPK pathway inhibitor PD169316, respectively. In conclusion, our findings demonstrated that *SIRT5* may modulate intramuscular fat deposition in goats through PI3k-Akt and MAPK signaling pathways. These results expand the gene regulatory network associated with IMF formation and provide a theoretical foundation for improving meat quality by targeting IMF deposition.

## Simple Summary

The aim of this study was to investigate the role of Silent Information Regulator 5 (*SIRT5*) in the regulation of lipid metabolism. We cloned the complete coding sequence of the goat *SIRT5* gene, analyzed its expression during preadipocyte differentiation, and investigated the effects of *SIRT5* gene expression on adipogenesis, cell proliferation, apoptosis, and lipid deposition in goat muscle preadipocytes. The main results of our study suggest that *SIRT5* plays a key role in regulating lipid utilization in goat preadipocytes through the PI3K-Akt and MAPK signaling pathways. These results provide valuable insights into the molecular mechanisms of intramuscular fat formation in goats and offer potential implications for improving meat quality by regulating intramuscular fat deposition.

## 1. Introduction

With the advancement of society, goat meat has emerged as a nutritionally valuable food source, characterized by its high protein content, low cholesterol levels, and abundance of beneficial fatty acids. This nutritional profile has led to increased consumer demand and subsequent research focus on the improvement of goat meat quality. Scientific evidence indicates that optimal levels of intramuscular fat (IMF) content are positively correlated with three crucial meat quality parameters: flavor enhancement, tenderness improvement, and juiciness optimization [1,2,3]. Although extensive research has identified multiple lipid metabolism genes involved in the complex process of muscular fat deposition, the specific molecular mechanisms governing intramuscular adipogenesis in ovine species remain incompletely understood and require further investigation.

Silent Information Regulator 5 (*SIRT5*), a member of the evolutionarily conserved Sirtuin (SIRT) family of lysine deacetylases (SIRT1-7) [4,5], plays a pivotal role in regulating redox homeostasis during mitochondrial respiration. *SIRT5* was first characterized by Frye and colleagues in 1999 from the testis Marathon cDNA library [6] and assumed to be primarily localized to mitochondria. However, increasing evidence indicates its additional presence in the cytosol [7] and wide involvement in glucose tolerance, accumulation of blood ammonia, lipid metabolism, and so on, although it may be dispensable for basal homeostasis [8]. The *SIRT5* gene encodes four distinct protein isoforms, with *SIRT5*iso1 being the most extensively characterized. A comprehensive investigation by Yipeng Du et al. systematically analyzed the subcellular localization, enzymatic activity, and tissue-specific distribution patterns of all four *SIRT5* isoforms in human cellular systems [9] largely by deacetylate, demalonylation, desuccinylation, and deglutarylation [8,10,11].

Current research has established that *SIRT5* expression and activity are regulated through the activation of the AMP-activated protein kinase (AMPK) signaling pathway under hypoxic conditions through increased AMP levels, which leads to subsequent phosphorylation and activation of peroxisome proliferator-activated receptor γ coactivator 1-α (PGC-1α). The activated PGC-1α then modulates *SIRT5* expression either directly or through the activation of peroxisome proliferator-activated receptor α (PPARα), establishing a complex regulatory network [12,13]. In addition, the expression of *SIRT5* was regulated by the direct interaction of miR-19b with the 3′-untranslated region (3′-UTR) of *SIRT5* mRNA [14]. As a critical regulatory protein, *SIRT5* exerts pleiotropic effects on numerous downstream targets, playing essential roles in various metabolic processes. Extensive research has demonstrated its involvement in the regulation of mitochondrial metabolism and insulin sensitivity [15,16], particularly through its modulation of key metabolic pathways including glycolysis, the tricarboxylic acid (TCA) cycle, fatty acid oxidation, nitrogenous waste management, and reactive oxygen species (ROS) detoxification [16]. The functional significance of *SIRT5* extends to various pathological conditions, particularly metabolism-related disorders. In mouse embryonic fibroblasts, *SIRT5* deficiency under nutrient starvation conditions leads to increased dynamin-related protein 1 (DRP1) levels, subsequently causing mitochondrial fragmentation and mitophagy [17]. SIRT5 knockout mice also exhibited less browning capacity in subcutaneous white adipose tissue and showed apparent cold intolerance [18] and may be associated with protein high-succinylation patterns, including glutamate dehydrogenase (GDH), alcohol dehydrogenase A (ADHA), and uncoupling protein 1 (UCP1), leading to impaired metabolic functions, manifested as glucose intolerance, defective cold adaptation, and mitochondrial dysfunction [19]. Additionally, *SIRT5* deficiency was shown to disrupt fatty acid metabolism, leading to abnormal accumulation of long-chain acylcarnitine in murine liver tissues [20].

Recent investigations have further expanded our understanding of *SIRT5*’s role in lipid metabolism. Hong et al. (2020) demonstrated that *SIRT5* inhibited lipid synthesis and deposition during bovine preadipocyte differentiation [21], with subsequent research revealing synergistic effects between *SIRT5* and *SIRT6* in regulating adipogenesis and lipid accumulation [22]. Complementary findings by Molinari et al. (2021) in 3T3-L1 adipocytes showed that *SIRT5* inhibition increased cellular triglyceride lipase activity and reduced lipid droplet size [23]. Despite these significant advances in understanding *SIRT5*’s regulatory functions in lipid metabolism across various species, its specific role in caprine lipid deposition remains unexplored, warranting further investigation.

## 2. Materials and Methods

### 2.1. Ethics Statement

All experimental exercises were reviewed and approved by the Institutional Animal Care and Use Committee, Southwest Minzu University (Chengdu, China). Permit number: S2020-013, revised in June 2004.

### 2.2. The Isolation, Culture, and Cryopreservation of Caprine Intramuscular Preadipocytes

The isolation of primary intramuscular preadipocytes from caprine tissue was performed according to established protocols with modifications [24]. Specifically, longissimus dorsi muscle tissues were aseptically collected from two two-day-old Jianzhou goats (randomly selected two unrelated two-day-old lambs) following humane euthanasia via exsanguination. Tissue samples were washed three times using phosphate-buffered saline (PBS) supplemented with 1% penicillin/streptomycin (*v*/*v*) and then cut up with sterilized ophthalmic scissors. For enzymatic digestion, tissue fragments were incubated with 0.2% type II collagenase (Sigma-Aldrich, St. Louis, MO, USA) at a ratio of 1 mL enzyme solution per gram of tissue, followed by gentle agitation at 37 °C for 90 min. The enzymatic reaction was terminated by adding an equal volume of DMEM/F12 medium (Gibco, Beijing, China) supplemented with 10% fetal bovine serum (FBS). The resulting cell suspension was sequentially filtered through sterile gauze and a 75 μm nylon mesh cell strainer. The filtrate was centrifuged at 2000 rpm/min for 5 min at room temperature, and the supernatant was carefully discarded. The cellular pellet was subsequently treated with erythrocyte lysis buffer for 5 min, followed by resuspension in complete growth medium (DMEM/F12 containing 10% FBS and 1% penicillin/streptomycin). The cell suspension was then transferred to 25 cm^2^ culture flasks and maintained at 37 °C in a humidified atmosphere containing 5% CO_2_. The culture medium was replaced every 48 h until cells reached approximately 80% confluence. Cells were passaged at a 1:3 ratio and cultured to the third generation before being seeded into 10 cm^2^ culture plates. For adipogenic differentiation, the growth medium was replaced with adipocyte induction medium consisting of MEM/F12 supplemented with 10% FBS, 1% antibiotic/antimycotic solution, and 50 μmol L^−1^ oleic acid (Sigma-Aldrich, Tokyo, Japan).

### 2.3. Cloning and Biological Analysis of the Goat SIRT5 Gene

Total RNA was extracted from various goat tissues (heart, liver, skin, lungs, kidneys, longissimus dorsi, large intestine, small intestine) using the Trizol method. Subsequently, cDNA was synthesized following the manufacturer’s protocol provided with the reverse transcription kit. The predicted sequence of the goat *SIRT5* gene, as published in GenBank (accession number: XM_018039221.1), was selected for further analysis. Specific primers (Forward: ATCCAGCGAGTCCATCTCAATT; Reverse: GCGCTGAGATTTCTTCCTTCC) were designed using Primer Premier 5.0 software [25]. The *SIRT5* gene sequence was subsequently cloned using genomic DNA extracted from the longissimus dorsi tissue of goats as the template.

Upon acquisition of the target gene sequences, a comprehensive genetic alignment of *SIRT5* gene sequences across various species was performed using the Blast program available through NCBI [26,27]. Subsequently, phylogenetic analysis was conducted to elucidate evolutionary relationships, utilizing MEGA5.0 [28] software for tree construction. In parallel, hydrophilicity analysis was performed through ProtScale (accessible at https://web.expasy.org/protscale/, accessed on 4 March 2025) to characterize the physicochemical properties of the SIRT5 protein.

### 2.4. Construction of Goat SIRT5 Gene Time Series Expression Profile

Fluorescence-specific primers for the target genes were designed using Primer Premier 5.0 software (primer sequences are provided in Supplementary Table S1). To ensure accurate quantification of gene expression, the UXT gene was employed as an endogenous control for normalization of relative expression levels. For comprehensive tissue-specific expression profiling, a total of 36 tissue samples (three randomly selected replicates from each of the 12 rams) were analyzed using quantitative reverse transcription PCR (qRT-PCR) to determine the spatial expression patterns of the *SIRT5* gene across multiple tissues in Jianzhou big-eared goats. Furthermore, to characterize the temporal dynamics of gene expression during adipocyte differentiation, intramuscular precursor adipocytes were harvested at specific time points (days 0, 1, 3, and 6) throughout the differentiation induction period for subsequent qRT-PCR analysis.

### 2.5. Vector Construction, Chemical Synthesis of siRNA, and Transfection

The pcDNA3.1(+) plasmid was subjected to double digestion using Hind III and Bam HI restriction enzymes, followed by ligation with the coding sequence (CDS) region of the *SIRT5* gene to construct the recombinant plasmid, designated as pcDNA3.1-*SIRT5*. The empty pcDNA3.1(+) plasmid served as a negative control and was designated as pcDNA3.1. Three small interfering RNA (siRNA) sequences targeting the goat *SIRT5* gene were designed (Table S1) and commercially synthesized by Gemma (Shanghai, China). The synthesized siRNAs were subsequently centrifuged at 12,000 rpm for 10 min and resuspended in 62.5 μL of diethylpyrocarbonate (DEPC)-treated water for further use.

For transfection experiments, intramuscular preadipocytes were seeded in 6-well plates and allowed to reach 70–80% confluence prior to transfection. The transfection procedure was performed following the manufacturer’s protocol for Lipofectamine™ 3000 reagent (Carlsbad, CA, USA), with each well receiving either 1 μg of plasmid DNA or 80 μM siRNA for transfection.

### 2.6. Oil Red O Staining and Triglyceride Assay

The cells cultured in 6-well plates were subjected to three washes with phosphate-buffered saline (PBS) and subsequently fixed with 4% formaldehyde at ambient temperature for 30 min. For Oil Red O staining, the working solution was prepared by mixing 3 mL of Oil Red O (5 g/L in isopropanol) with 2 mL of double-distilled water (ddH_2_O). This solution was then applied to the fixed cells and incubated at room temperature for 20 min. Following incubation, the cells were thoroughly rinsed with PBS and viewed using a microscope. To quantify the Oil Red O staining, 1 mL of isopropanol was added to each well of the 10 cm^2^ plate, and the absorbance at 510 nm was measured using a spectrophotometer (Thermo Fisher Scientific, Shanghai, China).

Intracellular triglyceride levels were determined using the Tissue Triglyceride (TG) Content Assay Kit (Applagin, E1013, Shanghai, China) according to the manufacturer’s protocol. Briefly, cells in the 10 cm^2^ plates were washed three times with PBS, followed by the addition of 200 μL of triglyceride lysis buffer. The cells were then incubated on ice for 10 min. The resulting lysate was divided into two aliquots: one for the BCA protein assay and the other for triglyceride quantification. Absorbance measurements were taken at 550 nm for triglycerides and at 562 nm for the BCA assay. The triglyceride concentrations were calculated based on a standard curve and normalized to the protein content determined by the BCA assay.

### 2.7. Total RNA Extraction and Quantitative Real-Time PCR (RT-qPCR)

Total RNA was isolated from goat intramuscular adipocytes using the RNAiso Plus reagent (Takara, 9109, Kusatsu, Japan) following the manufacturer’s protocol. RNA concentration and purity were assessed using a Nanodrop 2000 spectrophotometer (Thermo Fisher Scientific, Beijing, China), with all samples demonstrating absorbance ratios (260/280 nm) within the optimal range of 1.8 to 2.0. Subsequently, 1 μg of total RNA was reverse transcribed into complementary DNA (cDNA) using the Reverse Transcription Kit (VAzyme, R323-01, Nanjing, China).

Quantitative real-time PCR (qPCR) analysis was performed on a Bio-Rad CFX96 PCR System with Taq Pro Universal SYBR qPCR Master Mix (VAzyme, Q712-02, Nanjing, China) and gene-specific primers (Table S2). The ubiquitously expressed transcript (UXT) gene was employed as an internal reference for normalization. Relative gene expression levels were calculated using the comparative threshold cycle (2^−ΔΔCT)^ method.

### 2.8. RNA Sequencing (RNA-seq)

Goat intramuscular preadipocytes were cultured in 10 cm^2^ plates and subjected to differentiation induction for 48 h. Following differentiation, the cells were washed three times with sterile phosphate-buffered saline (PBS) and subsequently lysed with 1 mL of Trizol reagent for RNA extraction. The extracted RNA samples were then submitted to Hangzhou LC-Bio Technologies (Hangzhou, China)for transcriptome sequencing analysis. The resulting gene expression profiles obtained from RNA sequencing (RNA-seq) are presented in Supplementary Table S3. Differential gene expression analysis was performed using the DEseq2 package [29], with statistically significant differentially expressed genes (DEGs) identified based on the following criteria: adjusted *p*-value < 0.05 and absolute logFC > 2 (Supplementary Table S4). Functional annotation of the DEGs was conducted through Gene Ontology (GO) enrichment analysis (Supplementary Table S5) and Kyoto Encyclopedia of Genes and Genomes (KEGG) pathway analysis (Supplementary Table S6). Additionally, Gene Set Enrichment Analysis (GSEA) was performed using the goat gene set as the reference gene set to identify significantly enriched biological pathways.

### 2.9. Cell Counting Kit-8 (CCK-8) Assay

Cell proliferation of intramuscular adipocytes was assessed using the Cell Counting Kit-8 (CCK-8) assay. Cells were seeded in 96-well plates with three replicates for each experimental condition and transfected with either negative control siRNA (NC-*SIRT5*) or *SIRT5*-specific siRNA (SI-*SIRT5*), as well as empty vector (pcDNA3.1) or *SIRT5* overexpression vector (pcDNA3.1-*SIRT5*). At designated time points (0, 12, 24, 36, and 48 h post-transfection), 10 μL of CCK-8 reagent (AC11L054, Life-iLab, Shanghai, China) was added to each well, followed by incubation at 37 °C for 30 min. Finally, the optical density (OD) was measured at 450 nm using a microplate reader (Thermo Fisher Scientific, Carlsbad, CA, USA).

### 2.10. Flow Cytometry for Apoptosis Analysis

The apoptotic cell ratio was quantified using an Annexin V-FITC/PI Apoptosis Detection Kit (A211, Vazyme, Nanjing, China) following the manufacturer’s protocol. In brief, cells were seeded in six-well plates and transfected following the same procedure as described for the CCK-8 assay. Forty-eight hours post-transfection, cells were trypsinized and harvested. The cell suspension was resuspended in 100 µL of 1× binding buffer, followed by the addition of 5 µL Annexin V-FITC and 5 µL propidium iodide (PI). The samples were then incubated in the dark at room temperature for 15 min prior to flow cytometric analysis.

### 2.11. Western Blot Analysis

Total cellular proteins were extracted using RIPA lysis buffer (Solarbio Tech Inc., Beijing, China) supplemented with both protease inhibitor cocktail (04693132001, Roche, Mannheim, Germany) and phosphatase inhibitor. Protein samples were then separated by sodium dodecyl sulfate/polyacrylamide gel electrophoresis (SDS-PAGE) and subsequently transferred onto polyvinylidene fluoride (PVDF) membranes for immunoblotting analysis. The following primary antibodies were employed for protein detection: anti-β-actin (1:6000 dilution, BM0627, BOSTER, Wuhan, China), anti-phospho-AKT (1:2000 dilution, 4060, Cell Signaling Technology, Danvers, MA, USA), anti-AKT (1:1000 dilution, ab32505, Abcam, Cambridge, UK), anti-phospho-p38 MAPK (1:10,000 dilution, 3285S, Cell Signaling Technology, Danvers, MA, USA), and anti-p38 MAPK (1:10,000 dilution, 4, Cell Signaling Technology, Danvers, MA, USA). Protein bands were visualized using an enhanced chemiluminescence (ECL) detection system (Thermo Scientific, Waltham, MA, USA).

### 2.12. Statistical Analysis

Three independent biological samples were used for each experiment. Student’s t-test or one-way analysis of variance (ANOVA) was performed using SPSS version 22.0 (IBM Corp, Armonk, NY, USA). Duncan’s test was employed to determine significance, and values are presented as mean ± standard error of the mean (SEM). The data were visualized using GraphPad Prism v8.0 (GraphPad Software, La Jolla, CA, USA); ‘*’ indicates *p* < 0.05, ‘**’ indicates *p* < 0.01, and ‘***’ indicates *p* < 0.001.

## 3. Results

### 3.1. Cloning of Goat SIRT5 Gene and Its Temporal Expression Profile During Differentiation of Intramuscular Preadipocytes

**Figure 1:**
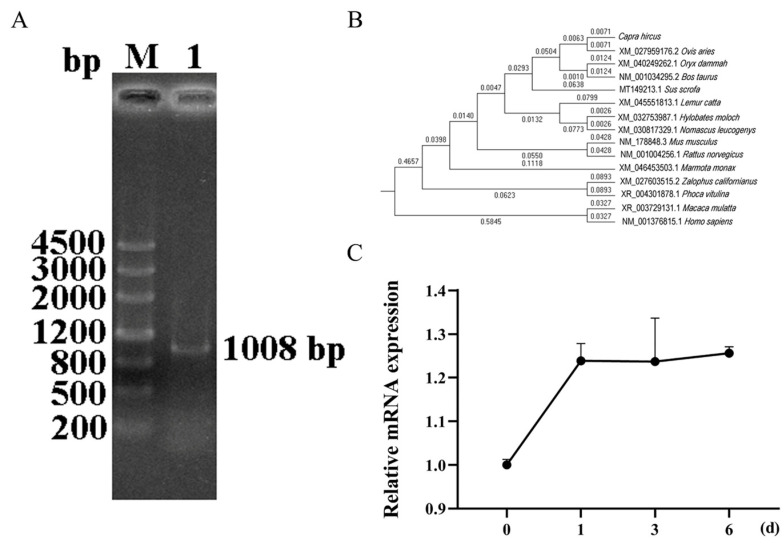
Molecular characterization and expression analysis of *SIRT5* in goats. (**A**) Electrophoretic analysis of the cloned goat *SIRT5* gene. Lane M: DNA Marker III; Lane 1: PCR product of the *SIRT5* gene. (**B**) Phylogenetic analysis of *SIRT5* across different species, demonstrating evolutionary relationships. (**C**) Temporal expression profile of *SIRT5* mRNA during adipocyte differentiation.

Primers were designed based on the predicted sequence of *Capra hircus SIRT5* (GenBank: XM_018039221.1) as listed in Table S2. Using cDNA synthesized from RNA extracted from the longissimus dorsi tissue of goats as a template, we amplified and cloned a 1008 bp nucleotide sequence of the goat *SIRT5* gene, including a 933 bp coding sequence (CDS) region flanked by a 45 bp 5’ untranslated region (UTR) and a 30 bp 3’ UTR, encoding a protein of 310 amino acid residues (Figure 1A). Following sequence acquisition, we conducted comprehensive bioinformatic analysis of the goat *SIRT5* gene. Comparative sequence analysis revealed high similarity with related species, showing 98.67% identity with *Ovis aries* (XM_027959176.2), 97.04% with *oryx dammah* (XM_040249257.1), and 96.73% with *Bos taurus* (NM_001034295.2). Phylogenetic analysis demonstrated that the goat *SIRT5* gene exhibits the closest evolutionary relationship with sheep, followed by oryx, cattle, and pig, while showing a more distant relationship with humans (Figure 1B). During adipocyte differentiation induction, quantitative analysis revealed a significant upregulation of *SIRT5* gene expression, reaching peak levels at day 1 and maintaining this elevated expression through day 6 (Figure 1C). These findings suggest that *SIRT5* maintains consistently high and stable mRNA expression levels throughout the differentiation process, indicating its potential crucial role in regulating cellular differentiation and maintaining lipid metabolism homeostasis.

### 3.2. Effect of Interference with the SIRT5 Gene on Lipid Deposition in Goat Intramuscular Precursor Adipocytes

**Figure 2:**
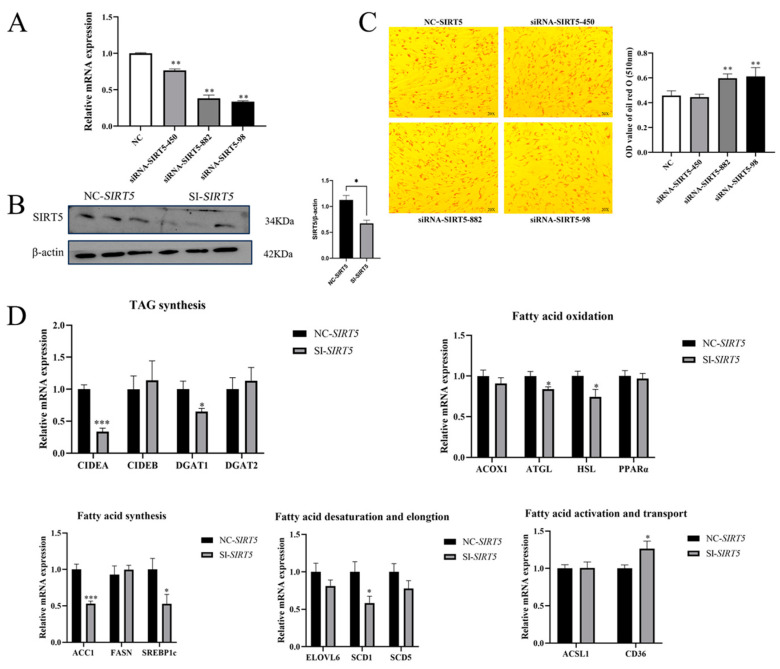
Effect of *SIRT5* gene interference on lipid deposition in goat precursor adipocytes. (**A**) *SIRT5* gene interference efficiency. (**B**) *SIRT5* protein expression after interference detected by WB. (**C**) Oil Red O staining and OD value of Oil Red O staining extraction after *SIRT5* (SI-*SIRT5*) interference (510 nm). (**D**) Effect of *SIRT5* gene interference on the expression levels of genes related to lipid metabolism. Data are shown as mean ± SEM, ‘*’ indicates *p* < 0.05, ‘**’ indicates *p* < 0.01, and ‘***’ indicates *p* < 0.001.

To investigate the functional role of *SIRT5* in lipid deposition regulation, we performed gain- and loss-of-function experiments by transfecting goat intramuscular progenitor adipocytes with either pcDNA3.1-*SIRT5* (overexpression) or Si-*SIRT5* (knockdown) constructs. Quantitative analysis revealed significant knockdown efficiencies of 33% (*p* < 0.01), 62% (*p* < 0.01), and 64% (*p* < 0.01) for siRNA-*SIRT5*-450, siRNA-*SIRT5*-882, and siRNA-*SIRT5*-98, respectively (Figure 2A). Western blot analysis further confirmed the protein-level knockdown efficiency of siRNA-*SIRT5*-98, demonstrating a significant reduction in SIRT5 protein expression (*p* < 0.05, Figure 2B). Based on these validation results, siRNA-*SIRT5*-98 was selected for subsequent functional studies.

To investigate the functional role of *SIRT5* in lipid accumulation within goat intramuscular progenitor adipocytes, we performed *SIRT5* gene knockdown experiments using specific siRNAs (SI-*SIRT5*) to establish loss-of-function models. Following *SIRT5* gene silencing, we systematically evaluated lipid droplet formation and analyzed the expression profiles of key genes involved in lipid metabolism. Our experimental results demonstrated differential effects across siRNA treatment groups: while the siRNA-*SIRT5*-450 group showed no statistically significant alteration in lipid deposition compared to the control group, both the siRNA-*SIRT5*-882 and siRNA-*SIRT5*-98 groups exhibited markedly enhanced lipid droplet accumulation (*p* < 0.01, Figure 2C). These findings reveal a positive correlation between the efficiency of *SIRT5* interference and the degree of lipid accumulation, indicating that *SIRT5* gene suppression facilitates lipid deposition in goat intramuscular progenitor adipocytes. To elucidate the impact of *SIRT5* deletion on lipid metabolism-related gene expression, we systematically analyzed the transcriptional profiles of key regulatory genes following *SIRT5* knockdown. As illustrated in Figure 2D, SIRT5 downregulation significantly upregulated the fatty acid transporter gene *CD36* (*p* < 0.05) while markedly suppressing the expression of the fatty acid desaturase gene *SCD1* (*p* < 0.01). Notably, the expression levels of other fatty acid desaturation-related genes (*SCD5*) and key fatty acid activation genes (*ACSL* and *ELOVL3*) remained unchanged in *SIRT5*-deficient cells. Furthermore, although the expression of the de novo fatty acid synthesis gene *FASN* showed no significant alteration, we observed a substantial reduction in its transcriptional regulator *SREBP1c* (*p* < 0.05). Regarding fatty acid oxidation pathways, *SIRT5* knockdown significantly decreased the expression of *ATGL* (*p* < 0.05) and *HSL* (*p* < 0.05) while maintaining stable expression levels of *ACOX* and its transcriptional regulator PPARα. In the context of triacylglycerol (TAG) metabolism, we detected a significant downregulation of *CIDEA* (*p* < 0.05), a gene associated with TAG secretion, whereas *CIDEB* expression remained unaffected. Additionally, the expression of *DGAT1* showed a significant reduction (*p* < 0.05), contrasting with the stable expression pattern observed for *DGAT2*.

### 3.3. Inhibition of Proliferation of Goat Intramuscular Precursor Adipocytes After Interference with the SIRT5 Gene

**Figure 3:**
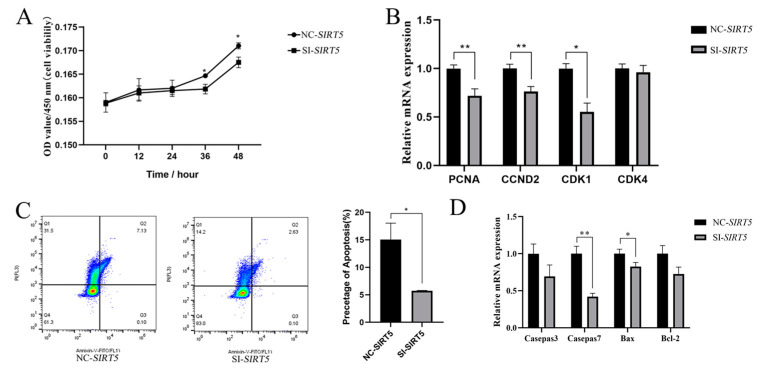
Effects of *SIRT5* gene silencing on proliferation and apoptosis of goat adipocytes. (**A**) *SIRT5* gene downregulation inhibited the proliferation of goat adipocytes, and the OD value of 450 nm was a characterization of cell viability. (**B**) Effects on the expression level of cell proliferation-related genes after *SIRT5* gene downregulation. (**C**) Effects of *SIRT5* gene downregulation on apoptosis of goat adipocytes. (**D**) Effects on the expression level of apoptosis-related genes after *SIRT5* gene downregulation. Data are shown as mean ± SEM, ‘*’ indicates *p* < 0.05, ‘**’ indicates *p* < 0.01.

To elucidate the regulatory role of *SIRT5* in the proliferation of goat intramuscular precursor adipocytes, we conducted comprehensive analyses using CCK-8 assays and flow cytometry. The CCK-8 viability assessment revealed a significant reduction in cellular activity in the *SIRT5* knockdown group compared to the control group (*p* < 0.05), particularly at 36 h and 48 h post-treatment (Figure 3A). Subsequent analysis of proliferation-related gene expression demonstrated that *SIRT5* downregulation significantly suppressed the mRNA levels of key cell cycle regulators, including *PCNA* (*p* < 0.01), *CCND2* (*p* < 0.01), and *CDK1* (*p* < 0.05) while maintaining stable expression of *CDK4* (Figure 3B).

Flow cytometry analysis further indicated that *SIRT5* suppression significantly attenuated apoptosis in goat adipocytes (*p* < 0.05, Figure 3C). Consistent with this observation, molecular analysis of apoptosis-related genes revealed that *SIRT5* downregulation markedly decreased the mRNA expression levels of *Caspase7* (*p* < 0.001) and *Bax* (*p* < 0.05), whereas the expression levels of *Caspase3* and *Bcl-2* remained unaffected (Figure 3D). These findings collectively suggest that *SIRT5* plays a crucial regulatory role in both proliferation and apoptosis pathways in goat intramuscular precursor adipocytes.

### 3.4. Effect of SIRT5 Gene Overexpression on Lipid Deposition in Goat Intramuscular Precursor Adipocytes

**Figure 4:**
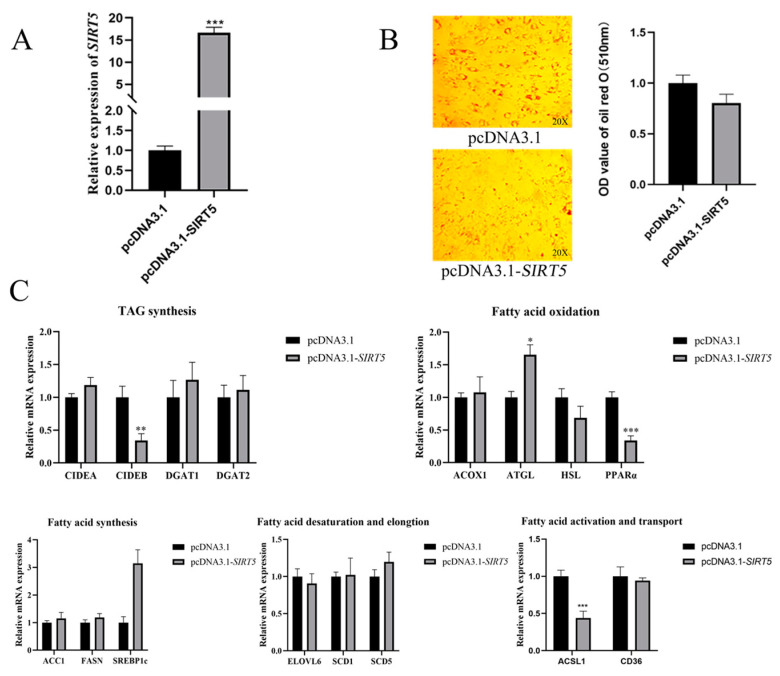
Effect of *SIRT5* gene overexpression on lipid deposition in goat precursor adipocytes. (**A**) Overexpression efficiency of *SIRT5* (pcDNA3.1-*SIRT5*). (**B**) Oil Red O staining and Oil Red O staining extracted OD (510 nm) after *SIRT5* (pcDNA3.1-*SIRT5*) overexpression. (**C**) Effect of *SIRT5* gene overexpression on lipid metabolism related gene expression levels. Data are shown as mean ± SEM, ‘*’ indicates *p* < 0.05, ‘**’ indicates *p* < 0.01, and ‘***’ indicates *p* < 0.001.

Our findings demonstrate that the downregulation of the *SIRT5* gene facilitates lipid accumulation in goat intramuscular precursor adipocytes. To further elucidate the role of *SIRT5* in lipid metabolism, we examined the effects of *SIRT5* upregulation on the expression of genes associated with lipid droplets and lipid metabolism. We engineered a pcDNA3.1-*SIRT5* construct to overexpress *SIRT5* and introduced it into goat intramuscular precursor adipocytes. Quantitative analysis revealed a 16-fold increase in *SIRT5* expression in the pcDNA3.1-*SIRT5* group compared to the negative control transfected with pcDNA3.1 (*p* < 0.01, Figure 4A). However, Oil Red O staining indicated that *SIRT5* overexpression did not significantly alter lipid droplet accumulation in these adipocytes (Figure 4B). To assess the impact of *SIRT5* overexpression on lipid metabolism-related gene expression, we analyzed the transcriptional levels of several key genes. As depicted in Figure 4C, *SIRT5* overexpression did not significantly affect the expression of *CD36*, *ELOVL3*, *SCD1*, or *SCD5*. However, a marked reduction was observed in the expression of *ACSL*, a critical gene involved in fatty acid activation (*p* < 0.01). Furthermore, while the expression of the *FASN*, *ACC1*, and *SREBP1c* genes central to de novo fatty acid synthesis remained largely unchanged, a notable increase in *SREBP1c* expression was detected. In terms of fatty acid oxidation, ATGL expression was significantly upregulated (*p* < 0.05), whereas *ACOX1* and *HSL* levels remained stable. Conversely, the transcriptional regulator *PPARα* was significantly downregulated (*p* < 0.001). Additionally, the expression of *CIDEA*, *DGAT1*, and *DGAT2*, genes associated with TAG secretion, showed no significant changes, but *CIDEB* expression was significantly reduced (*p* < 0.05). These results suggest that *SIRT5* plays a complex role in regulating lipid metabolism in goat intramuscular precursor adipocytes, influencing specific pathways without broadly altering lipid droplet accumulation.

### 3.5. Promotion of Proliferation of Goat Intramuscular Precursor Adipocytes After Overexpression of the SIRT5 Gene

**Figure 5:**
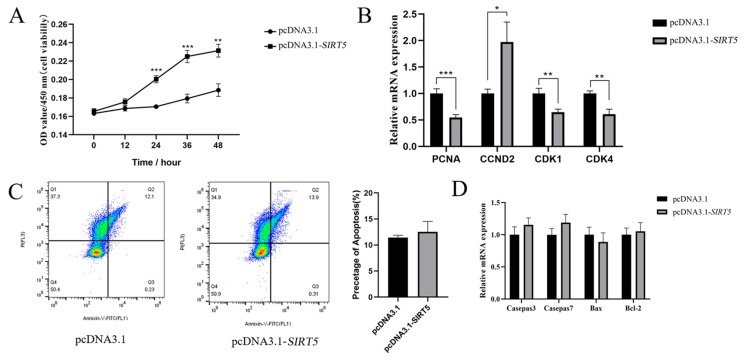
Effect of overexpressing the *SIRT5* gene on the proliferation and apoptosis of goat adipocytes. (**A**) Upregulation of the *SIRT5* gene inhibited the proliferation of goat adipocytes, and the OD value of 450 nm was a characterization of cell viability. (**B**) Effect of upregulation of *SIRT5* gene on the expression level of genes related to cell proliferation. (**C**) Effect of upregulation of *SIRT5* gene on the apoptosis of goat adipocytes. (**D**) Effect of upregulation of effect on the expression level of apoptosis-related genes after upregulation of *SIRT5* gene. Data are shown as mean ± SEM, ‘*’ indicates *p* < 0.05, ‘**’ indicates *p* < 0.01, and ‘***’ indicates *p* < 0.001.

To investigate the functional role of *SIRT5* in cellular proliferation, we conducted a series of experiments following our initial observation that *SIRT5* gene interference inhibits cell proliferation. To determine whether *SIRT5* overexpression promotes cell proliferation, we assessed cell viability with the CCK-8 assay and flow cytometry analysis. The experimental results revealed a statistically significant enhancement in cell viability in the pcDNA3.1-*SIRT5* group compared to the pcDNA3.1 control group at three time points: 24 h (*p* < 0.01), 36 h (*p* < 0.01), and 48 h (*p* < 0.05, Figure 5A). In parallel, we examined the expression profiles of proliferation-associated genes in adipocytes overexpressing *SIRT5*. Quantitative analysis demonstrated that *SIRT5* upregulation resulted in a marked reduction in the expression levels of *PCNA* (*p* < 0.001), *CDK1* (*p* < 0.01), and *CCND2* (*p* < 0.01) genes while simultaneously inducing a significant increase in *CCND2* expression (*p* < 0.05, Figure 5B). To further characterize the biological effects of *SIRT5* overexpression, we evaluated its impact on adipocyte apoptosis using flow cytometry. The quantitative analysis indicated that *SIRT5* overexpression did not significantly influence the apoptotic rate of goat adipocytes (Figure 5C). Consistent with these findings, subsequent examination of apoptosis-related gene expression profiles showed that *SIRT5* overexpression failed to substantially alter the expression levels of key apoptotic markers, including *Casepas7*, *Casepas3*, *Bax*, and *Bcl-2* (Figure 5D). These comprehensive findings suggest that while *SIRT5* overexpression significantly enhances cellular viability and modulates the expression of proliferation-related genes, it does not appear to exert substantial effects on adipocyte apoptosis or the expression of apoptosis-related genes under the experimental conditions employed in this study.

### 3.6. Interfering with FATP4 to Alter Gene Expression Profiles in Goat Precondition Adipocytes

**Figure 6:**
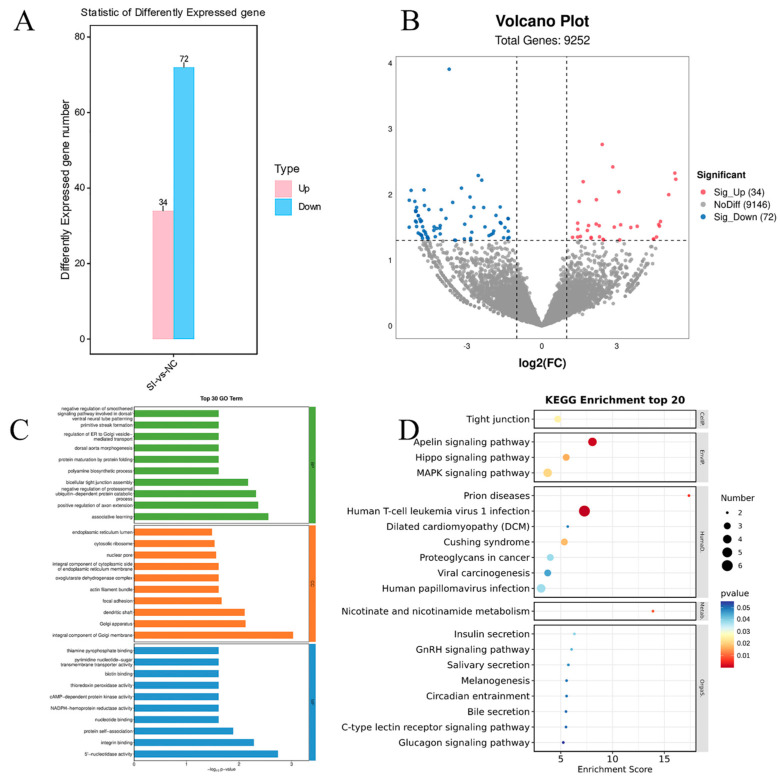
Analysis of DEGs after interfering with the *SIRT5* gene. (**A**) Bar graph of the number of differential genes; red indicates upregulated genes and blue indicates downregulated genes (*p* < 0.05, logFC > 2). (**B**) Volcano graph of the counted differential genes; pink colors indicate upregulated genes, and blue indicates downregulated genes (*p* < 0.05, logFC > 2). (**C**) Interfering with the GO of the *SIRT5* gene enrichment analysis; (**D**) DRG analysis after interfering with *SIRT5* gene.

To elucidate the molecular mechanisms underlying lipid deposition promotion through *SIRT5* gene interference, we conducted sequencing and comprehensive analysis of total RNA transcriptomes from both SIRT5-interfered goat adipocytes and negative controls. As illustrated in Figure 6A,B, our analysis identified 106 differentially expressed genes (DEGs) meeting the significance thresholds (*p* < 0.05, logFC > 2). The DEG profile revealed 72 downregulated genes and 34 upregulated genes. Subsequent Gene Ontology (GO) enrichment analysis, presented in Figure 6C, demonstrated that the DEGs were significantly enriched across three major categories: (1) biological processes, particularly in cellular processes, metabolic processes, bioregulation, bioprocess regulation, and cellular component organization or biosynthesis in response to stimuli; (2) cellular components, with predominant enrichment in cell parts, organelles, organelle parts, and membranes; and (3) molecular functions, primarily involving binding, catalytic activity, and molecular structure regulation. To further investigate the signaling pathways associated with these DEGs, we performed KEGG pathway analysis. The results, depicted in Figure 6D, revealed significant enrichment of DEGs in several critical pathways, including focal adhesion, cancer-related proteoglycans, the MAPK signaling pathway, the glycolysis/gluconeogenesis signaling pathway, pathways in cancer, and the Hippo signaling pathway. These findings provide valuable insights into the complex regulatory networks involved in lipid deposition following *SIRT5* interference.

### 3.7. The SIRT5 Gene Promotes Lipid Deposition in Goat Intramuscular Precursor Adipocytes via the PI3k-Akt and p38 Signaling Pathways

**Figure 7:**
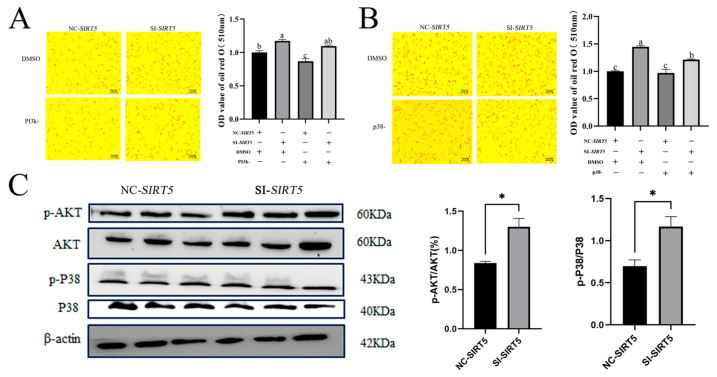
Interference with *SIRT5* gene promotes lipid deposition in goat premise adipocytes through PI3K-Akt and MAPK signaling pathways. (**A**) The promotion of cellular lipid deposition by interference with *SIRT5* is dependent on the PI3K-Akt pathway. (**B**) The promotion of cellular lipid deposition by interference with *SIRT5* is dependent on the MAPK pathway. (**C**) Western blot detection of the changes in the levels of p-AKT/AKT and p-p38/p38 proteins after overexpression and interference with p-AKT/AKT and p-p38/p38 protein levels after *SIRT5*. Data are shown as mean ± SEM, ‘*’ indicates *p* < 0.05. Different lower case letters indicate significant differences.

The RNA-seq analysis revealed significant enrichment of the MAPK signaling pathway in the KEGG enrichment analysis of differentially expressed genes (DEGs), with additional identification of the PI3K-Akt signaling pathway in Table S6. These findings suggest that *SIRT5*-mediated promotion of lipid deposition in goat precursor adipocytes may be regulated through both MAPK and PI3K-Akt signaling pathways. To investigate this hypothesis, we employed DMSO as a negative control and conducted inhibitor treatments using LY294002 (PI3K pathway inhibitor) and PD169316 (p38 MAPK pathway inhibitor). The experimental results demonstrated distinct pathway-specific responses: while the SI-*SIRT5* group showed no significant changes in lipid deposition following PI3K inhibition (LY294002 treatment) compared to the NC group, a marked inhibition of lipid deposition was observed in the SI-*SIRT5* group upon p38 MAPK pathway inhibition (PD169316 treatment), as illustrated in Figure 7A,B. To further validate these findings at the protein level, we examined the expression patterns of key signaling molecules in *SIRT5*-interfered goat intramuscular precursor adipocytes. Specifically, we quantified the levels of total and phosphorylated forms of AKT and p38 proteins. As shown in Figure 7C, the SI-*SIRT5* group exhibited significantly elevated ratios of p-AKT/AKT (%) and p-p38/p38 compared to the NC group (*p* < 0.05), indicating enhanced pathway activation. These collective results demonstrate that *SIRT5*-mediated regulation of lipid deposition in goat precursor adipocytes is modulated through both PI3K-Akt and p38 MAPK signaling pathways, with the p38 pathway playing a particularly significant role in this regulatory mechanism.

## 4. Discussion

*SIRT5*, a widely expressed protein in animals, plays a crucial regulatory role in various post-translational modifications, including but not limited to acetylation, malonylation, succinylation, and glutarylation. This multifunctional enzyme is actively involved in several critical cellular metabolic pathways, such as glycolysis, the tricarboxylic acid (TCA) cycle, and fatty acid oxidation, thereby significantly contributing to essential cellular biological processes. Despite its well-documented functions in these fundamental metabolic activities, the specific role of *SIRT5* in regulating intramuscular fat deposition in goats remains poorly understood and warrants further investigation. The present study is novel in its pioneering systematic investigation into the regulatory mechanism of the *SIRT5* gene in intramuscular fat deposition in goats. Through comprehensive molecular cloning of the goat *SIRT5* gene and subsequent construction of its spatiotemporal expression profile, we identified consistently high expression levels of *SIRT5* during the early stages of adipocyte differentiation. Experimental manipulation revealed that *SIRT5* knockdown significantly enhanced lipid deposition while concurrently inhibiting cellular proliferation. Conversely, *SIRT5* overexpression did not markedly influence lipid accumulation but demonstrated a proliferative effect. Utilizing RNA sequencing (RNA-seq) coupled with functional validation experiments, we have, for the first time, elucidated that *SIRT5* modulates lipid metabolic homeostasis through its regulatory influence on both the PI3K-Akt and MAPK signaling pathways. Furthermore, our findings demonstrate that *SIRT5* indirectly regulates lipid droplet accumulation via the glycolysis and fatty acid oxidation pathways. These findings not only address a critical knowledge gap regarding *SIRT5*’s role in the regulation of intramuscular fat deposition in goat muscle but also establish a substantial theoretical foundation for molecular-level optimization of goat meat quality.

The present study revealed that *SIRT5* maintains consistently high mRNA expression levels throughout the differentiation process of goat intramuscular adipocytes from day 1 to day 6. This expression pattern contrasts markedly with that observed in bovine precursor adipocytes, where *SIRT5* expression peaks on the first day of differentiation and subsequently declines, returning to baseline levels (day 0) by day 11 [21]. Furthermore, comparative analysis of different adipocyte models demonstrates distinct *SIRT5* expression dynamics: in 3T3-L1 preadipocytes, *SIRT5* expression exhibits a biphasic pattern characterized by initial upregulation followed by gradual downregulation, whereas in brown preadipocytes, a progressive increase in *SIRT5* expression is observed throughout differentiation. The functional significance of *SIRT5* in adipocyte differentiation is further supported by pharmacological and genetic evidence, in which experimental administration of MC3482, a specific *SIRT5* inhibitor, was shown to promote brown adipose tissue formation during differentiation [23]. Complementary to these findings, Shuai et al. [18] demonstrated that *SIRT5* knockdown led to significant impairment of brown adipocyte differentiation. Collectively, these findings suggest that despite the cell-type-specific expression patterns of *SIRT5* during adipocyte differentiation, this protein plays an essential regulatory role, particularly in the early stages of adipocyte differentiation.

In this study, following the cloning of the goat *SIRT5* gene, we explored the regulatory effects of *SIRT5* on intramuscular fat (IMF) deposition using RNA silencing and overexpression techniques. Our findings indicate that silencing *SIRT5* significantly increases lipid deposition in goat intramuscular precursor adipocytes. Conversely, overexpression of *SIRT5* did not result in significant changes, although a decreasing trend was observed. This aligns with previous findings that *SIRT5* inhibits lipid deposition in bovine precursor adipocytes [21] and reduces lipid droplet accumulation in mice [30]. Further analysis revealed that silencing of *SIRT5* significantly upregulated the expression of the *CD36* gene while downregulating that of *ATGL1* and *HSL*. In contrast, *SIRT5* overexpression led to a significant decrease in *ACSL1* and *PPARα* gene expression and a significant increase in *ATGL1* expression. These results suggest that *SIRT5* may play a moderately significant role in regulating fatty acid oxidation [31], with a lesser impact on the rate of fatty acid synthesis. The absence of *SIRT5* has been shown to reduce fatty acid oxidation and lead to the accumulation of long-chain fatty acyl CoAs in the heart [10]. Similarly, the deletion of *SIRT5* in mouse liver results in the accumulation of long-chain fatty acid carnitine [32]. Previous studies have also highlighted that the primary pathways regulated by *SIRT5*-containing malonylated proteins are glycolysis and gluconeogenesis. Overexpression of *SIRT5* in mouse liver is associated with increased glycolytic flux and enhanced fatty acid oxidation [33], whereas *SIRT5* knockdown significantly reduces glycolytic flux in primary hepatocytes [7]. Consistent with these findings, our RNA-seq results demonstrated enrichment in the glycolytic pathway following *SIRT5* gene interference. In conclusion, based on the aforementioned results, we hypothesize that *SIRT5* may regulate lipid accumulation in goat intramuscular precursor adipocytes through the glycolysis signaling pathway, where fatty acid oxidation is attenuated, thereby influencing lipid accumulation in these cells.

Intramuscular fat (IMF) content is primarily determined by the number and size of intramuscular adipocytes, with the processes of apoptosis and proliferation playing critical roles in regulating these factors [34]. In this study, we observed that silencing *SIRT5* inhibited the proliferation of goat intramuscular precursor adipocytes, a finding consistent with the results reported by Liang et al. in hepatocellular carcinoma [35]. Conversely, overexpression of *SIRT5* in goat precursor adipocytes promoted cell proliferation, aligning with the findings of Peng et al. in human colorectal cancer [36]. Similarly, *SIRT5* overexpression enhanced the proliferation of hepatocellular carcinoma cells, while *SIRT5* knockdown significantly suppressed their proliferation [37]. These collective findings underscore the role of *SIRT5* in regulating cell proliferation across different cell types. Apoptosis is another critical factor influencing cell numbers. Our study revealed that silencing *SIRT5* inhibited apoptosis in goat intramuscular precursor adipocytes, with a decreased ratio of the apoptosis-related genes *Bax* to *Bcl-2* and reduced expression of the *Caspase7* gene. This observation is consistent with the findings of Wen Gu et al., who identified *SIRT5* as a key regulator of autophagy and apoptosis in gastric cancer cell lines, noting that *SIRT5* is downregulated during apoptosis [38]. Additionally, Rixin Zhang et al. reported that *SIRT5* knockdown significantly inhibited apoptosis in hepatocellular carcinoma cells [37], further supporting our results. Interestingly, overexpression of *SIRT5* did not significantly affect apoptosis in goat intramuscular precursor adipocytes, nor did it induce notable changes in the expression of apoptosis-related genes. Therefore, the precise mechanisms by which *SIRT5* overexpression influences apoptosis warrant further investigation. In summary, our findings suggest that *SIRT5* plays a dual role in regulating both proliferation and apoptosis in goat intramuscular precursor adipocytes, with its silencing inhibiting both processes. However, the impact of *SIRT5* overexpression on apoptosis remains unclear and requires additional exploration to elucidate its underlying mechanisms.

To elucidate the mechanisms by which *SIRT5* gene interference promotes lipid droplet accumulation, we conducted RNA sequencing (RNA-seq) to analyze alterations in cellular mRNA levels following *SIRT5* suppression. Subsequent KEGG and GO analyses revealed that differentially expressed genes were predominantly enriched in signaling pathways associated with the immune system. This observation aligns with previous studies demonstrating the involvement of SIRT family members in the pathogenesis of various diseases [39,40]. Notably, our analysis also identified significant enrichment of genes in lipid metabolism-related pathways, including fatty acid degradation, MAPK signaling, glycolysis/gluconeogenesis, and PI3K-Akt signaling pathways. Among these signaling cascades, the PI3K-AKT pathway emerges as a pivotal intracellular signaling axis that orchestrates lipid metabolism through phosphorylation-dependent regulation of downstream effectors [41,42]. The PI3K-AKT signaling axis serves as a central regulator of lipid metabolic gene expression, exerting systemic control over key metabolic organs including the liver [43], skeletal muscle [44], brain [45], and adipose depots [46]. The PI3K-AKT signaling pathway exerts bidirectional control over lipid homeostasis by transcriptionally regulating key genes involved in both anabolic [47] and catabolic pathways [48], thereby orchestrating systemic lipid metabolism. The MAPK signaling cascade serves dual regulatory functions, orchestrating cellular proliferation while concurrently modulating lipid metabolic homeostasis [49]. The MAPK signaling cascade comprises four principal branches: (1) the canonical Ras–Raf–extracellular signal-regulated kinase (ERK1/2) pathway [50], (2) the ERK5 (BMK1) pathway [51], (3) the p38 MAPK pathway [52], and (4) the c-Jun N-terminal kinase (JNK) pathway [50]. These distinct yet interconnected signaling modules mediate specific cellular responses to extracellular stimuli.

To further investigate these findings, we performed functional validation experiments using goat intramuscular precursor adipocytes. Following *SIRT5* interference, cells were treated with specific inhibitors targeting the PI3K pathway (LY294002) and p38 MAPK pathway (PD169316) [34,53]. The experimental results demonstrated a marked reduction in lipid metabolism upon inhibitor treatment. Western blot analysis concurrently revealed increased phosphorylation levels of AKT and p38 following *SIRT5* interference. These findings are consistent with previous research by Hong et al., who reported that *SIRT5* inhibits bovine preadipocyte differentiation and lipid deposition through AMPK activation and MAPK signaling pathway inhibition [21]. Furthermore, *SIRT5* has been shown to synergistically interact with SIRT6 in regulating bovine preadipocyte differentiation and lipid metabolism via the AMPKα signaling pathway [22]. Based on these collective findings, we propose a mechanistic model wherein *SIRT5* regulates lipid deposition in goat intramuscular precursor adipocytes through modulation of the PI3K-Akt and MAPK signaling pathways. This hypothesis provides a potential molecular framework for understanding the role of *SIRT5* in lipid metabolism regulation.

This study primarily investigated the mRNA-level changes to elucidate that differentially expressed genes following *SIRT5* interference are predominantly enriched in disease-related pathways, particularly cancer, while potentially regulating lipid deposition in goat intramuscular precursor adipocytes through glycolysis/gluconeogenesis, PI3K-Akt, and MAPK signaling pathways. However, we recognize that *SIRT5* is known to regulate post-translational modifications (PTMs) of numerous cellular proteins, and the impact of *SIRT5* gene expression on protein modification levels remains unexplored in our current investigation. Specifically, the identification of which protein PTMs in goat intramuscular precursor adipocytes are significantly influenced by altered *SIRT5* expression, and through which specific modifications intramuscular fat deposition is regulated, remains to be determined. These aspects represent notable limitations in the present study.

## 5. Conclusions

Through in vitro cloning, we successfully isolated the *SIRT5* gene and established that the alterations in *SIRT5* expression significantly influence the redistribution of lipid metabolism between intramuscular fat (IMF) deposition and cellular proliferation in goat intramuscular precursor adipocytes. Moreover, our experimental results provide compelling evidence that *SIRT5* modulates intramuscular lipid deposition in goats through the regulation of PI3k-Akt and MAPK signaling pathways. These findings substantially advance our understanding of the functional role of *SIRT5* in lipid metabolism regulation and provide mechanistic insights into its potential regulatory networks governing intramuscular adipogenesis in goats. Meanwhile, these findings may help us to better understand the role of *SIRT5* in regulating lipid deposition in goats using CRISPR/Cas9 technology.

## Data Availability

The datasets generated for this study can be found in the NCBI Bio Project database (https://www.ncbi.nlm.nih.gov/bioproject, accessed on 4 March 2025); the mRNA-Seq accession number is PRJNA1231255.

## Supplementary Materials

The following supporting information can be downloaded at: https://www.mdpi.com/article/10.3390/ani15071072/s1; the Supplementary Materials contain documents Table S1 (siRNA sequences of *SIRT5*), Table S2 (Primers for quantitative real-time PCR (RT-qPCR)), Table S3 (ALL_FPKM), Table S4 (Differential genes were screened using DEsq2 at *p* < 0.05 and logFC > 2), Table S5 (GO enrichment analysis), and Table S6 (KEGG enrichment analysis).
